# A propos d'un cas de syndrome de Sweet

**DOI:** 10.11604/pamj.2013.14.120.2375

**Published:** 2013-03-28

**Authors:** Fadwa El Amrani, Badredine Hassam

**Affiliations:** 1 Service de Dermatologie, CHU Ibn Sina, Université Med V, Souissi, Rabat, Maroc

**Keywords:** Syndrome de Sweet, dermatose aiguë, dermatose neutrophilique

## Image en médicine

Le syndrome de Sweet (SS) ou dermatose aiguë fébrile neutrophilique est la dermatose neutrophilique la plus fréquente. Il est caractérisé par une fièvre, une éruption de plaques érythémateuses, surélevées et douloureuses, un syndrome inflammatoire et un infiltrat dermique massif constitué essentiellement de PNN. Il est souvent idiopathique, mais peut parfois s'associer à diverses affections notamment aux hémopathies malignes. La corticothérapie constitue le traitement de référence mais d'autres molécules sont également efficaces notamment l'indométacine. Nous rapportons le cas d'un patient de 84 ans, sans antécédents pathologiques, qui consulte pour une éruption cutanée douloureuse, d'installation brutale précédée une semaine auparavant par un syndrome pseudo-grippal. L'examen clinique retrouvait un patient fébrile à 38,5°C, qui présente des plaques papulo-nodulaires érythémato-violines, à surface mamelonnée, pseudo-vésiculeuse, siégeant au dos des mains, au visage et à la nuque. Une hyperhémie conjonctivale bilatérale a été notée. Le bilan biologique a révélé un syndrome inflammatoire avec une hyperleucocytose à 16100/mm3 faite de 13000/mm3 de PNN, une vitesse de sédimentation accélérée à 52 mm et une CRP à 253 mg/l. La biopsie cutanée était en faveur d'un SS en montrant un infiltrat dermique superficiel fait de PNN sans vascularite associée. Le bilan étiologique à la recherche d'une maladie inflammatoire, d'une connectivite ou d'une néoplasie associée était négatif. Le diagnostic de SS médicamenteux a été écarté devant l'absence de prise médicamenteuse. Le diagnostic de SS idiopathique a été alors retenu. Le malade a été mis sous indométacine à 100 mg/j avec une nette amélioration des symptômes en une semaine.

**Figure 1:**
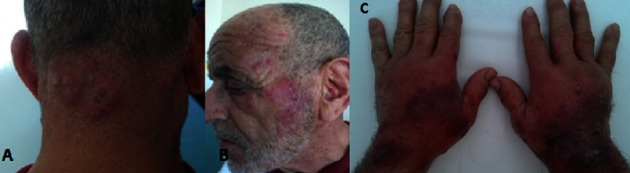
Lésions papulo-nodulaires érythémato-violines de la nuque (A), du visage (B) et à surface pseudo-bulleuse au dos des mains (C)

